# Real‐World Persistence and Characteristics of Type 2 Diabetes Patients Prescribed Semaglutide in Scotland

**DOI:** 10.1111/1753-0407.70102

**Published:** 2025-05-21

**Authors:** David Alexander Dickie, Ramil Burden, Alexander C. Miller, Lucy Mackillop, Kevin Heath, Sumit Dutta, Jesse Dawson

**Affiliations:** ^1^ Optum Eden Prairie Minnesota USA; ^2^ School of Cardiovascular and Metabolic Health, College of Medical, Veterinary & Life Sciences University of Glasgow, Queen Elizabeth University Hospital Glasgow UK; ^3^ Nuffield Department of Women's & Reproductive Health University of Oxford, John Radcliffe Hospital Oxford UK

**Keywords:** persistence, real‐world, semaglutide, type 2 diabetes

## 1 Introduction

People taking glucagon‐like peptide‐1 receptor agonists (GLP‐1s), such as semaglutide, have achieved clinically meaningful weight loss (≥ 5%) in large clinical trials over 24 months [1, 2]. Weight loss is an important aspect of the management of type 2 diabetes [3]. Using health records from the NHS Greater Glasgow and Clyde Safe Haven (https://www.nhsggc.scot/staff‐recruitment/staff‐resources/research‐and‐innovation/nhsggc‐safe‐haven/), we aimed to explore real‐world persistence with initiated 1 mg/0.74 mL 3 mL semaglutide prefilled injection pens and associated body mass index (BMI) changes among type 2 diabetes patients in Scotland.

## 2 Results

There were 37 984 prescriptions for semaglutide (1 mg/0.74 mL 3 mL) prefilled injection pens dispensed to 2293 unique patients with type 2 diabetes between August 2019 and February 2024. Mean patient age was 57.3 ± 11.2 years and 1139 (49.7%) were female. The single largest patient group was middle aged females (33.7%). Most patients (69.5%) were white, and a large majority were from lower socioeconomic backgrounds (73.5%).

Out of 1568 patients with a first semaglutide prescription dispensing date at least 2 years before the end of the reporting period (February 2024), 935 (59.6%) were persistent at 24 months.

Changes in BMI by measurement interval are shown in the Table 1. Twenty‐five percent of patients with > 3‐month measurement intervals achieved improvement in BMI category, 27% with > 6‐month measurement intervals, 28% with > 12‐month measurement intervals, and 31% with > 24‐month measurement intervals.

**TABLE 1:** Changes in BMI by measurement time.

Measurement interval	Baseline BMI (kg/m^2^)	End BMI (kg/m^2^)	BMI change (kg/m^2^)	Test statistics
> 3‐month	34.71 ± 6.06	33.83 ± 6.18	−0.89 ± 2.53	*t* = −10.11, *p* < 0.001
> 6‐month	34.60 ± 6.00	33.67 ± 6.13	−0.93 ± 2.61	*t* = −9.73, *p* < 0.001
> 12‐month	34.51 ± 5.65	33.51 ± 5.85	−1.00 ± 2.59	*t* = −9.11, *p* < 0.001
> 24‐month	34.32 ± 5.17	33.28 ± 5.36	−1.04 ± 2.62	*t* = −6.32, *p* < 0.001

## 3 Comment

We found that a large majority of type 2 diabetes patients prescribed semaglutide in Scotland were from lower socioeconomic backgrounds. Sixty percent of patients persisted with semaglutide at 24 months. Statistically significant reductions in BMI (~1 kg/m^2^) were observed during measurement intervals from > 3 to > 24 months. Approximately one third of patients achieved improvement in BMI category over > 24 months. These changes are lower than reported in previous clinical trials [2] and may reflect the healthcare challenges people from lower socioeconomic backgrounds face in the real world.

More research is required to set GLP‐1 pricing models reflective of real‐world efficacy and persistence. There should be an assessment of services that can support patients with type 2 diabetes, particularly those from lower socioeconomic backgrounds, to persist with and maximize the benefits of semaglutide and other GLP‐1 s.

## Author Contributions

D.A.D., R.B., A.C.M., and J.D. conceived and designed this work; all authors contributed to its interpretation. D.A.D. and J.D. acquired and analyzed the data presented. D.A.D. wrote the initial draft and all authors contributed to editing and review of this manuscript and approved the final version for publication.

## Conflicts of Interest

D.A.D., R.B., A.C.M., L.M., K.H., and S.D. are employees of Optum, a provider of healthcare technology, pharmacy care and direct healthcare services. J.D. has no conflicts of interest to report.
